# Raffinose Capped Silver Nanoparticles: A New Localized Surface Plasmon Resonance Based Sensor for Selective Quantification of Cr(VI) in Waste Waters

**DOI:** 10.3390/molecules26175418

**Published:** 2021-09-06

**Authors:** Penka Vasileva, Lubomir Djerahov, Irina Karadjova

**Affiliations:** 1 Faculty of Chemistry and Pharmacy, University of Sofia “St. Kliment Ohridski”, 1, J. Bourchier Blvd., 1164 Sofia, Bulgaria; Karadjova@chem.uni-sofia.bg; 2 Department of Chemistry, University of Mining and Geology “St. Ivan Rilski”, Students Town, “Prof. Boyan Kamenov” Street, 1700 Sofia, Bulgaria; lubomirdjerahov@mgu.bg

**Keywords:** colorimetric sensor, plasmonic, chromium(VI), silver nanoparticles, raffinose coating

## Abstract

In this study, a new method for selective determination of Cr(VI) in water samples at pH 4 is presented using raffinose capped silver nanoparticles (Ag/Raff NPs) as an optical sensor. The method is based on the variation of LSPR absorption band intensity as a result of electrostatic interaction between the negatively charged Ag/Raff NPs and positive Cr(III) ions, in-situ produced by chemical reduction of Cr(VI) with ascorbic acid, combined with the fast kinetics of Cr(III) coordination to the –OH groups of the capping agent on the nanoparticle surface, further causing the nanoparticle aggregation. The calibration curve for Cr(VI) is linear in the range 2.5–7.5 μmol L^−1^, the limit of quantification achieved is 1.9 μmol L^−1^, and values of relative standard deviation vary from 3 to 5% for concentration level 1.9–7.5 μmol L^−1^. The interference studies performed in the presence of various metal ions show very good selectivity of Ag/Raff NPs toward Cr(VI) species. The added–found method is used to confirm the accuracy and precision of developed analytical approach.

## 1. Introduction

Chromium is a relatively abundant element in the earth’s crust, existing predominantly as Cr(III) compounds. The Cr(VI) compounds are also found in nature at much lower quantities as a result of natural oxidation of Cr(III) in the presence of manganese minerals. The biological activity of Cr(III) and Cr(VI) species, their chemical behavior and toxic effects are quite different: Cr(III) is nontoxic and involved in several biochemical processes such as metabolism of glucose and lipids, reactions of enzymes, body fat decrease; Cr(VI) is toxic to most living organisms, causing lung cancer, dermatitis, allergies, kidney damage [1]. The industrial application of Cr(VI) compounds is still very high, leading to release of toxic Cr(VI) species and calls for strict analytical control of Cr(III) and Cr(VI) content in wastes, waste waters and other environmental matrices. That is why the environmental quality standards and individual permissible limits for waste waters, which have been introduced through European and national legislations, require reliable speciation analysis of Cr or at least selective quantification of concentrations of highly toxic Cr(VI) species. In addition, taking into account high oxidation power and chemical activity of Cr(VI) as well as its lower concentrations in comparison with Cr(III) species, the analytical procedures developed should ensure fast determination of Cr(VI) on site, during the sampling process. From such a point of view, the direct methods developed for Cr(VI) quantification are preferable and of particular interest. Several review papers have been published on modern analytical strategies for Cr speciation in various matrices [2,3,4,5,6,7]. Expensive and time consuming hyphenated methods, such as ion chromatography or capillary electrophoresis in combination with ICP-MS measurement provide accurate results for both Cr species, but simple and fast nonchromatographic procedures for targeted determination of only Cr(VI) are still useful for routine laboratory practice [8,9]. The optical sensing and determination of Cr(VI) using nanomaterials is an excellent approach which ensures low detection limits and good precision; at the same time, the sensing methods developed are rapid, low-cost and can be easily read out with the naked eye. Recently, excellent reviews for colorimetric detection of metal ions (e.g., Hg(II), Pb(II), Cu(II)) using noble metal nanoparticles have been reported, based on different response mechanism [10,11]. Silver and gold nanoparticles functionalized or unmodified have been already reported as colorimetric sensors for both Cr(III) and Cr(VI) [12,13,14,15,16,17,18,19,20,21,22,23,24]. The predominant concept for Cr(III) determination has been based on the aggregation of noble metals nanoparticles which results in a color change and the appearance of a new secondary band in response to the surface plasmon absorption of aggregated nanoparticles [12,13,14,15,16,17,18,19,20]. The complexation of Cr(III) with the capping agent of nanoparticles or with specific ligands used for nanoparticle modification has been suggested as an initial step for the cross-linking of nanoparticles [14,15,16]. The selectivity of analytical probes developed for Cr(III) sensing is questionable. The literature search showed conclusions for highly selective procedures; however, also application of additional reagents such as EDTA or preliminary treatment of sample with carbonate is suggested for removal of observed interferences of coexisting ions [13,25]. Conventionally, Cr(VI) detection has been achieved after reduction of Cr(VI) with a suitable reductant [12,13,14,15,16,21,22,23]. As a rule, the speciation of both species is made in two steps: the first is selective determination of Cr(III) and the second is determination of total Cr after reduction of Cr(VI) in a parallel sample [14,15,16]. Hence, the toxic Cr(VI) existing at a lower concentration level is quantified through a subtraction of two relatively high values, which leads to high uncertainty of the results achieved.

In the past years, some non-aggregation colorimetric sensors for selective detection of Cr(VI) based on redox etching/leaching of AuNPs or AgNPs have been developed [26,27,28,29]. Very recently, a surface-enhanced Raman spectroscopy-based analytical procedure for selective quantification of Cr(VI) even in the presence of higher quantities of Cr(III) has been proposed [30].

In the present study, optimal conditions for direct and selective determination of Cr(VI) in the presence of Cr(III) are proposed by using raffinose capped silver nanoparticles as an LSPR optical sensor. The nanoparticles used were prepared by a green synthesis method utilizing raffinose as both reducing and capping agent. Careful kinetic studies and optimization of the response time of the optical sensor ensured selective, accurate and reliable determination of Cr(VI) in waters in only 5 min. A calibration method for Cr(VI) quantification, based on systematic study of matrix interferences, is proposed. The analytical approach developed is suitable for routine application and might be used also as a fast screening method on site. Analytical figures of merit, the validation method and application to real samples are presented.

## 2. Results and Discussion

Selective colorimetric determination of Cr(VI) in the presence of Cr(III) in water samples is based on the variation of LSPR absorption band intensity of Ag/Raff NPs as a result of electrostatic attraction between the negatively charged nanoparticles and the positive Cr(III) ions, produced in situ by chemical reduction of Cr(VI) with ascorbic acid, combined with fast kinetics of Cr(III) coordination to the –OH groups of the capping agent on the nanoparticle surface, causing further the nanoparticle aggregation.

### 2.1. Nanoparticle Characterization

#### 2.1.1. Optical Characteristics and Stability of Nanoparticle Aqueous Dispersion at Storage

**Figure 1:**
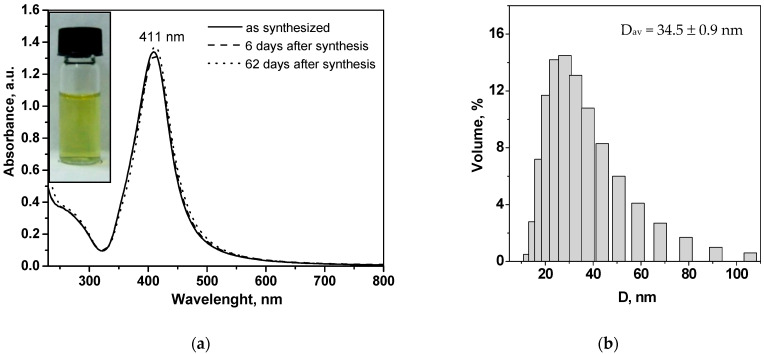
(**a**) UV-Vis absorption spectra of raffinose capped silver nanoparticles taken after nine-fold dilution of the aqueous dispersions immediately after synthesis, after one week, and after 2 months of storage (inset: digital photograph); (**b**) Particle size distribution histogram of raffinose capped silver nanoparticles obtained by dynamic light scattering (DLS) measurements.

Experimental UV-Vis spectra of diluted aqueous dispersions of the synthesized raffinose capped AgNPs in the wavelength range 200–800 nm are reported in Figure 1a.

The spectrum of freshly synthesized raffinose capped silver nanoparticles (solid line in Figure 1a) shows a single, narrow and high-intensive absorption band with a maximum wavelength of 411 nm, known as Localized Surface Plasmon Resonance (LSPR, characteristic of the collective absorption of free electrons in the conduction band of silver nanoparticles), which indicates the formation of mainly spherical nanosized silver particles with a narrow size distribution [31]. In addition, the high stability of AgNPs is confirmed through the UV–Vis spectra of nanoparticle aqueous dispersion, recorded after different periods of aging (one week and two months). The spectra, presented in Figure 1a with dashed lines, are superimposable with that recorded immediately after the preparation, showing a high stability of raffinose capped silver nanoparticles, synthesized via environmentally friendly procedure described (see Section 3.2).

Figure 1b shows the particle size distribution histogram of raffinose capped AgNPs recorded using dynamic light scattering (DLS) technique. The average hydrodynamic nanoparticle diameter obtained from DLS analysis is 34.5 nm, while the zeta potential value given by zeta sizer analysis is 47.2 ± 1.1 mV at pH 6.8 (in the presence of 0.001 mol L^−1^ KCl). As reported in the literature [32], an absolute value of zeta potential in the range from 30 to 60 mV indicates a good stability of nanoparticles in aqueous dispersion, which is consistent with the results from UV-Vis spectroscopy, presented in Figure 1a. This can be attributed to the high electrostatic stabilization in addition to the steric nanoparticle stabilization due to the layer of raffinose molecules on the nanoparticle surface. The negative value of ξ-potential is most likely due to the sorption of negatively charged oxidized forms of raffinose fragments, resulting from the alkaline raffinose degradation in the course of nanoparticle synthesis [33].

#### 2.1.2. Morphology and Crystal Structure

**Figure 2:**
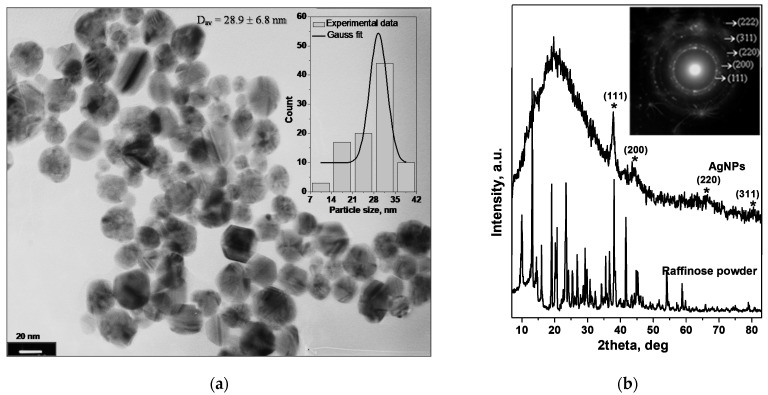
(**a**) TEM micrograph of raffinose capped silver nanoparticles (inset: size distribution histogram); (**b**) X-ray diffraction patterns of raffinose powder and layer of raffinose capped silver nanoparticles, dried on glass support (inset: Selected area electron diffraction (SAED) pattern obtained from TEM observations), “*”—diffraction peaks of Ag/Raff NPs.

The UV-Vis spectrum based conclusion, concerning the formation of silver nanoparticles with narrow size distribution and approximately spherical morphology, is further confirmed by TEM observations (Figure 2a).

The TEM micrograph and size distribution histogram illustrate quasi-spherical silver nanoparticles with narrow size distribution and average diameter of 28.9 ± 6.8 nm. In addition to the nanospheres, some typical polyhedral nanoparticles (multiple twined nanocrystals) and nanorods with small aspect ratios are also observed in the presented TEM image. The diffused rings in the selected area electron diffraction (SAED) pattern (inset in Figure 2b) confirmed the polycrystalline nature of the synthesized raffinose capped silver nanoparticles.

It is obvious that the value of the hydrodynamic nanoparticle diameter mentioned above (34.5 ± 0.9 nm) is larger than that obtained by TEM observation (28.9 ± 6.8 nm). This discrepancy can be related both to the molecules adsorbed on the nanoparticle surface (e.g., stabilizers) and to the thickness of the electrical double layer (solvation).

The crystallinity of raffinose capped silver nanoparticles was further confirmed by XRD analysis. The XRD pattern of Ag/Raff NPs (Figure 2b) shows four relatively broad diffraction peaks with 2*θ* of 38.2°, 44.4°, 64.6°, and 77.4° corresponding to the (111), (200), (220), and (311) planes, respectively, of the face-centered cubic (fcc) silver (PDF 04-0783). The halo at reflection angles in the range 20–30° is due to the glass support, used in the sample preparation for XRD analysis. No impurity peaks were observed in the X-ray diffraction pattern of silver nanoparticles synthesized. The relatively broad diffraction peaks indicate either the imperfect or fine nanocrystallite nature of the polycrystalline Ag/Raff NPs [34]. Furthermore, the characteristic diffraction reflections of raffinose are not observed in the diffractogram of raffinose capped silver nanoparticles, most probably due to the formation of non-crystalline complexes of the silver nanoparticles with raffinose [35]. The results from XRD analysis are consistent with the SAED pattern obtained in TEM observations.

### 2.2. Raffinose Capped Silver Nanoparticles as LSPR Based Optical Sensor for Cr(VI) Determination

#### 2.2.1. Optimization of Experimental Procedure

Several parameters were investigated systematically in order to establish optimal conditions for the selective colorimetric detection of Cr(VI), in situ reduced to Cr(III) by ascorbic acid (AA) in the presence of raffinose capped silver nanoparticles.

As a first step, the pH value was adjusted, taking into account the analysis of real samples and their preservation. The acidity of the medium is an important parameter that determines the behavior of both Ag/Raff NPs and analyte. In an acidic environment, pH below 3, silver nanoparticles are characterized with relatively low stability because of their aggregation followed by oxidative dissolution. With increasing pH (above pH 4.5), silver nanoparticles became more stable, unlike the Cr(III) species, freshly produced by Cr(VI) reduction, due to their hydrolysis at pH above 4.5. The experiments performed showed that final pH about 4 ensured the highest sensitivity and could be accepted as an optimal sample medium. This acidity was obtained as a natural result of the following optimal volume ratios in which the aqueous dispersion of raffinose capped silver nanoparticle (with pH about 7), standard solution of Cr(VI) (with pH 3.8 achieved by hydrochloric acid), and ascorbic acid solution were mixed:

*V*(Ag/Raff NPs, mL):*V*(Cr(VI), mL, pH 3.8):*V*(AA, mL) = 0.20:1.65:0.15 (1)

**Figure 3:**
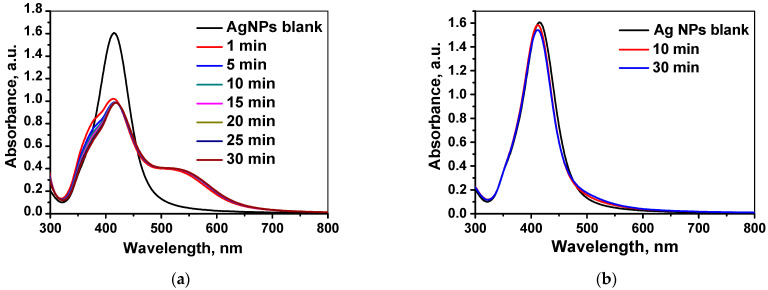
Evolution of UV-Vis absorbance spectrum of Ag/Raff NPs (**a**) upon addition of 5 × 10^−5^ mol L^−1^ Cr(VI), in situ reduced to Cr(III) by ascorbic acid at pH 4, and (**b**) upon addition of 5 × 10^−5^ mol L^−1^ Cr(III) at pH 4.

As a second step, the kinetics of interaction between raffinose capped silver nanoparticles and Cr(III), in situ obtained by chemical reduction of 5 × 10^−5^ mol L^−1^ Cr(VI) with ascorbic acid at final pH 4 was studied within half an hour using UV-Vis spectroscopy. Typical evolution of UV-Vis absorbance spectrum with time is shown in Figure 3a. For comparison, the kinetics of interaction between Ag/Raff NPs and 5 × 10^−5^ mol L^−1^ Cr(III) was also followed under the same experimental conditions and presented in Figure 3b. It is seen that the sensor’s response (absorption intensity of LSPR band of Ag/Raff NPs at λ_max_) toward Cr(III), in situ obtained by Cr(VI) reduction, is significant during the first 5 min of contact time. On the contrary, in the presence of Cr(III), the change of absorption intensity of Ag/Raff NPs band at λ_max_ is negligible not only during the first 5 min, but also throughout the period of observation (30 min). This experimental fact allows selective detection of Cr(VI) species even in the presence of Cr(III) if the sensor’s response is measured within the first 5 min of contact time. This difference in the response kinetics of the developed optical probe, Ag/Raff NPs, toward Cr(III) as standard aqueous solution on one hand, and toward Cr(III), in situ obtained by reduction of Cr(VI) with ascorbic acid on the other hand, could be explained by the well-known extremely high inertness of the [Cr(H_2_O)_6_]^3+^ complex, existing in the aqueous solutions of Cr(III). According to the proposed scenario, Cr(III) is most probably coordinated to the raffinose –OH groups on the surface of AgNPs, thus inducing their aggregation and change of absorption band intensity at λ_max_. The kinetics of this complexation process is extremely slow if Cr(III) is already blocked as inert [Cr(H_2_O)_6_]^3+^ complexes. In the presence of raffinose capped silver nanoparticles, Cr(III) ions, freshly produced by chemical reduction of Cr(VI) with ascorbic acid, immediately involve in complex formation with –OH groups of raffinose inducing aggregation of Ag/Raff NPs in the first 5 min of contact time. The data showed above indicate that a critical parameter for the selective determination of toxic chemical species Cr(VI) using raffinose capped silver nanoparticles as an optical probe is the contact time between the sensor nanoparticles and analyte species, and its optimal value is 5 min.

#### 2.2.2. Analytical Characteristics of the Developed LSPR Based Optical Sensor for Selective Quantification of Cr(VI)

**Figure 4:**
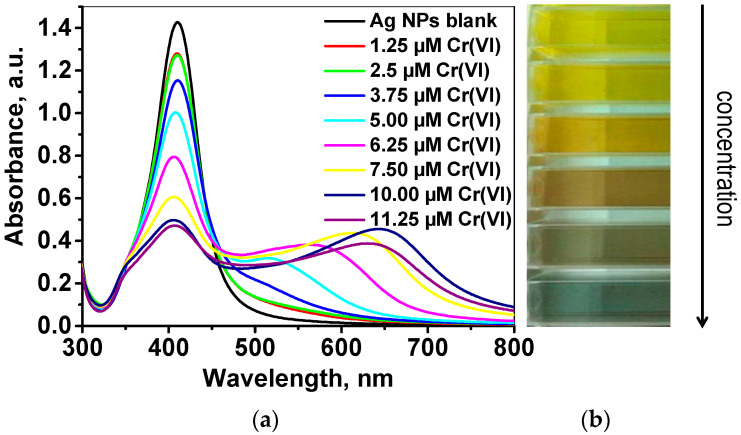
(**a**) UV-Vis absorption spectra and (**b**) color changes of Ag/Raff NPs recorded 5 min after addition of Cr(VI) standard solutions with increasing concentrations, in situ reduced with ascorbic acid at pH 4.

The sensitivity and applicability of raffinose capped silver nanoparticles for quantitative determination of Cr(VI) were studied under the defined optimal conditions. The colorimetric response and LSPR band behavior was monitored as a function of Cr(VI) concentrations in the range 1.0–11.5 µmol L^−1^ (see experimental details described in Section 3.4). Briefly, into a mixture of 200 μL as-synthesized Ag/Raff NPs and 150 μL 5 × 10^−3^ mol L^−1^ ascorbic acid, placed in a quartz cuvette, a volume of 1650 μL standard solution of Cr(VI) at pH 3.8 was added until final Cr(VI) concentration in the range from 1.0 to 11.5 µmol L^−1^ and final pH about 4. The resulting mixture was stirred with Vortex for 30 s, and after a contact time of 5 min, the UV-Vis absorption spectrum was recorded in the wavelength range 300–800 nm and presented in Figure 4a, along with a change in the color of Ag/Raff NPs dispersion (Figure 4b).

A gradual decrease of the intensity of characteristic plasmon band at 411 nm is observed with an increase of Cr(VI) concentration. In addition, a shoulder band has appeared at the wavelength range 550–650 nm, whose intensity increases along with decreasing intensity and a slight blue shift of the main plasmon band at 411 nm. This phenomenon is already reported and described as a change of the refractive index of the particles as a result of sorption of the positive Cr(III), produced by Cr(VI) reduction, onto the negatively charged nanoparticle surface [36]. Most probably, the distance between the negatively charged silver nanoparticles is reduced as a result of complexation between the freshly produced positive Cr(III) and the surface –OH groups of raffinose capped silver nanoparticles, leading to subsequent partial nanoparticle aggregation. The degree of aggregation depends proportionally on the concentration of reduced Cr(VI).

**Figure 5:**
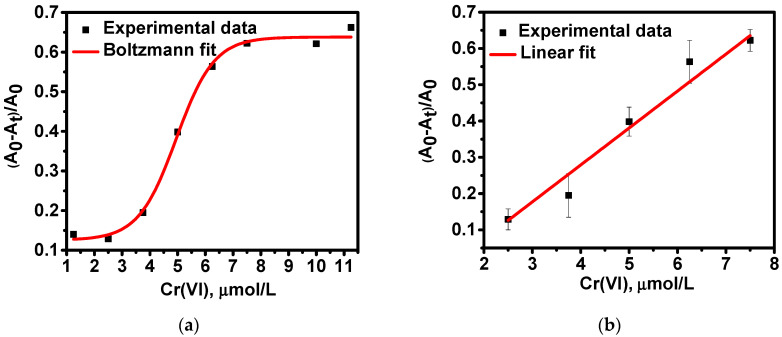
(**a**) Optical response of LSPR based sensor using raffinose capped silver nanoparticles as a function of Cr(VI) concentrations in the range 1.0–11.5 µmol L^−1^ and (**b**) calibration curve in the concentration range 2.5–7.5 μmol L^−1^ Cr(VI). R^2^ = 0.9798.

For quantitative determination of Cr(VI), the change of LSPR band intensity of raffinose capped silver nanoparticles at 411 nm upon addition of analyte solutions with various concentrations was estimated as a ratio (*A*_0_ − *A*_t_)/*A*_0_, where *A*_0_ corresponds to the intensity of absorbance maximum of blank Ag/Raff NPs (without addition of Cr(VI)) and *A*_t_ corresponds to the intensity of absorbance maximum of Ag/Raff NPs after addition of Cr(VI) standard solutions at the optimized conditions described above (Figure 5a). A linear correlation exists between the relative change of absorbance maximum intensity Δ*A*_r_ = (*A*_0_ − *A*_t_)/*A*_0_ and the concentration of Cr(VI) over the concentration range 2.5–7.5 μmol L^−1^ (Figure 5b):

Δ*A*_r_ = 0.1019 × *c*(Cr(VI)) − 0.1287 (2)

It can be concluded that the developed LSPR based sensor for quantification of Cr(VI) using raffinose capped silver nanoparticles as an optical probe has a linear response for Cr(VI) concentrations in the range 2.5–7.5 μmol L^−1^ with a limit of quantification (LOQ) of 1.9 μmol L^−1^, calculated using standard deviation of measurements at 2.5 µmol L^−1^, derived equation between the relative change of absorbance maximum intensity Δ*A*_r_ = (*A*_0_ − *A*_t_)/*A*_0_ and the concentration of Cr(VI) applying 10σ criterion. Additional experiments have been performed aiming to confirm statistically significant difference between LOQ and 2.5 µmol L^−1^ (initial point of linear range). Although the dependence between the absorbance change and Cr(VI) concentration is not linear in the range between 1.9 and 2.5 µmol L^−1^, still, quantitative determination is possible at this lowest concentration range. The calculated LOD (3σ criterion) using the same standard deviation is 0.65 µmol L^−1^. The values of relative standard deviation vary from 3% to 5% for the concentration range 1.9–7.5 μmol L^−1^ Cr (VI).

#### 2.2.3. Selectivity of the Developed Optical Sensor

**Figure 6:**
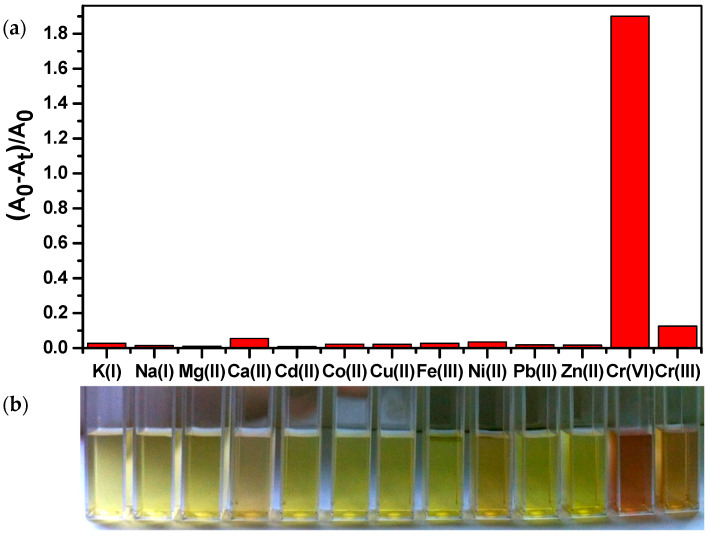
(**a**) Relative change of absorption band intensity of raffinose capped silver nanoparticles with the addition of different kinds of metal ions, including K(I), Na(I), Mg(II), Ca(II), Cd(II), Co(II), Cu(II), Fe(III), Ni(II), Pb(II), Zn(II), Cr(III) (concentration level at 50 μmol L^−1^), and Cr(VI) (concentration level at 5 μmol L^−1^) within a contact time of 5 min; (**b**) Digital images of corresponding mixtures.

From an analytical point of view, it is very important to define the selectivity of the proposed optical probe for colorimetric determination of Cr(VI). This has been evaluated through testing the response of raffinose capped silver nanoparticles to various environmentally relevant metal ions including K(I), Na(I), Mg(II), Ca(II), Pb(II), Cu(II), Zn(II), Cd(II), Fe(III), Co(II), and Ni(II) under the same conditions as in the case of Cr(VI) (see Section 3.4). The optical response of Ag/Raff NPs to the tested ions (concentration level at 50 μmol L^−1^) within a contact time of 5 min after their addition (separately for each ion) to Ag NPs dispersion is illustrated in Figure 6a. For comparison, the optical response of Ag/Raff NPs to Cr(III) at the same concentration level is also presented. It is easy to observe that all metal ions produce much weaker signals (almost at baseline level), associated with minor-to-negligible relative changes of absorption band intensity of raffinose capped silver nanoparticles. This selectivity is easily visualized even by the naked eye, as can be seen from the respective digital photos in Figure 6b.

The observed selectivity of the optical response of raffinose capped silver nanoparticles towards Cr(VI) is achieved because of optimally selected combination of two key parameters in the procedure: in situ reduction of Cr(VI) with ascorbic acid in the presence of raffinose capped silver nanoparticles and optimal contact time of 5 min between the analyte and the optical probe. 

### 2.3. Analytical Application

**Figure 7:**
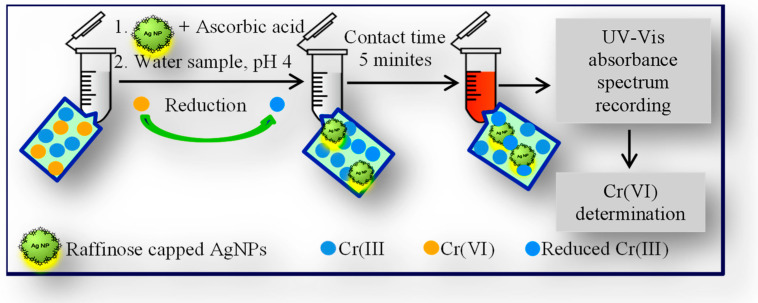
Scheme of analytical procedure for Cr speciation in surface waters using raffinose capped silver nanoparticles as efficient optical sensor for quantification of Cr(VI).

An analytical procedure, schematically presented in Figure 7, for Cr speciation in surface waters was developed based on fast screening for Cr(VI) content using raffinose capped silver nanoparticles as a selective and sensitive optical sensor (see experimental details described in Section 3.5).

To evaluate the practical application of developed optical sensor for quantification of Cr(VI), three types of experiments were performed. As a first step, the selectivity of Cr(VI) determination in the presence of 5–10 fold excess of Cr(III) was studied in model solutions prepared in distilled water. The results obtained are listed in Table 1 and undoubtedly verified selectivity and accuracy of Cr(VI) quantification even in the presence of 10 fold excess of Cr(III).

**Figure 8:**
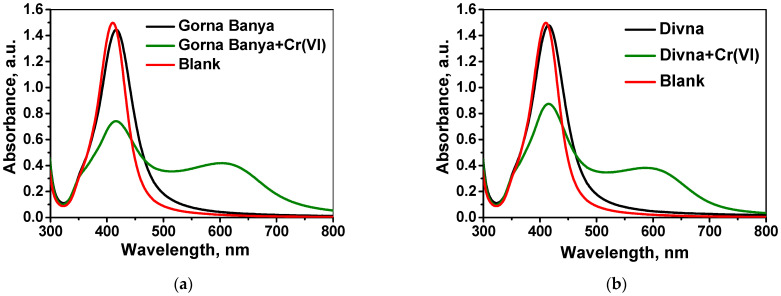
Response of the optical sensor to natural water samples without addition and with addition of standard solution of Cr(VI): (**a**) Gorna Banya mineral water; (**b**) Divna spring water.

As a second step, the accuracy and reproducibility of developed analytical procedure was examined by added–found method applied for Cr(VI) sensing in river and mineral waters with known concentrations of total Cr and spiked with known concentrations of Cr(VI). Two natural water samples, Gorna Banya mineral water and Divna spring water, were spiked with Cr(VI) at concentration level of 5 µmol L^−1^. The analysis of each of aqueous samples was performed according to the described procedure (Section 3.5), accompanied by analysis of blank sample (without addition of Cr(VI)). UV-vis absorption spectra, recorded in the wavelength range 300–800 nm, are shown in Figure 8.

It might be concluded that when adding an equal amount of standard solution of Cr (VI) to each of the studied matrices, the intensity of the surface plasmon absorption band at λ_max_ changes in varying degrees. The explanation could be the influence of the sample matrix on the complexation of Cr(VI) in real waters. For this reason, the standard addition calibration method is proposed for Cr(VI) quantification in real water samples. The obtained values for Cr(VI) content in the studied waters are below the LOD for the respective matrix 2.31 µg L^−1^ for Gorna Banya mineral water and 2.68 µg L^−1^ for Divna spring water. In order to confirm this conclusion, additional experiments based on the added–found method were performed with different water samples spiked with known amounts of Cr(VI). A calibration curve constructed with standards prepared in distilled water was initially used for Cr(VI) quantification in these samples; however, as already observed, the sample matrix affects the intensity of the sensor response, calling for a method of standard addition for calibration. The results obtained are summarized in Table 1 and confirmed accuracy and good reproducibility of the proposed sensor method for Cr(VI) determination.

Finally, the analytical procedure was validated by comparative analysis of underground waters, polluted with Cr(VI) from chromium plating factory, with the standard method based on spectrophotometry using diphenylcarbazide (ISO 11083: Water Quality —Determination of Chromium(VI)—Spectrometric Method Using 1,5-Diphenyl- carbazide). Very good agreement between the results obtained (Table 2, Student *t*-test, 95% confidence limit) undoubtedly validates the analytical probe developed (raffinose capped silver nanoparticles) for selective quantification of Cr(VI) and confirms the applicability of the developed analytical procedure for routine application in the laboratory practice.

A serious advantage of the proposed analytical method is the simple and environmentally friendly synthesis of Ag/Raff NPs, and the possibility to prepare a stock solution of silver nanoparticles with well controlled concentration, thus ensuring reproducible results between different batches. Indeed, parallel experiments, performed with raffinose capped silver nanoparticles from different batches as an optical sensor for determination of Cr(VI) in ground waters, show RSD values between 4 and 7%. Reliable results for Cr(VI) content were obtained within 5 min using only as-synthesized Ag/Raff NPs in a small volume of water sample—the whole procedure might be performed on site, during sampling and used for fast screening of ground waters from polluted regions. Additionally, the detection limits achieved are enough low to enable monitoring for Cr(VI) according to national and European legislation.

## 3. Materials and Methods

### 3.1. Chemicals and Materials

Analytical grade silver nitrate (AgNO_3_, 99.8%, Merck, KGaA, Darmstadt, Germany), D-(+) raffinose pentahydrate (Alfa Aesar, Karlsruhe, Germany), and sodium hydroxide (NaOH, 99%, Merck, Germany) were used to prepare aqueous dispersion of silver nanoparticles. The stock standard solutions of 500 mg L^−1^ Cr(VI) and 500 mg L^−1^ Cr(III) were prepared using K_2_Cr_2_O_7_ (Sigma-Aldrich, GmbH, Sternheim, Germany) and CrCl_3_·6H_2_O (Merck, Germany), respectively. Working standard solutions for Cr(VI) within the concentration range of 1 × 10^−6^–1 × 10^−4^ mol L^−1^ were prepared daily by appropriate dilution of the stock standard solution using 1.5 × 10^−3^ mol L^−1^ HCl. All diluted Cr(VI) solutions were kept refrigerated at 4 °C. Analytical grade ascorbic acid (AA) and salts of the different cations studied (NaCl, KCl, MgCl_2_, CaCl_2_, Pb(NO_3_)_2_, ZnCl_2_, CuCl_2_, NiCl_2_, CdCl_2_, CoCl_2_, FeCl_3_) were purchased from Merck, Germany. Doubly distilled water was used for preparation of all reagent solutions.

### 3.2. Synthesis of Raffinose Capped Silver Nanoparticles

**Figure 9:**
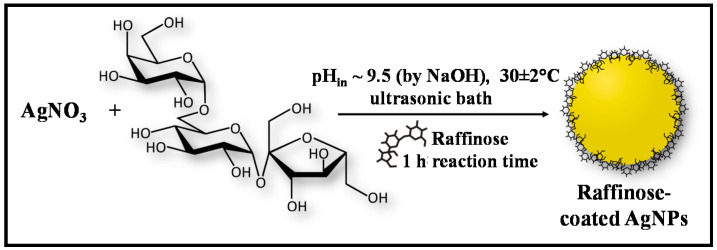
Schematic illustration of the procedure employed for green synthesis of raffinose capped silver nanoparticles.

The reaction was carried out in a sonication bath at an operating power output of 100 W and a frequency of 38 kHz (UST2.4–150, Siel, Gabrovo, Bulgaria). As already highlighted in our previous work, the main advantages of this ultrasound-mediated synthesis compared with the conventional reactions performed under mechanical or magnetic stirring are the improved reaction kinetics and enhanced chemical reactivity due to the acoustic cavitation, along with homogenizing the solution and maintaining the uniform concentration profile [35]. The synthesis of raffinose capped silver nanoparticles follows a green synthesis protocol as described in our previous study [37], but on an enlarged scale. A schematic representation of the applied procedure is shown in Figure 9.

Ag/Raff NPs were obtained by one-step, one-phase “green” synthesis based on chemical reduction of Ag^+^ using D-(+) raffinose as both reducing and capping agent, and sodium hydroxide as a reaction catalyst. Briefly, 0.75 mL 0.1 mol L^−1^ AgNO_3_ solution were added to 60.75 mL double distilled water and homogenized in an ultrasonic bath for 5 min. Thereafter, 7.5 mL 0.1 mol L^−1^ raffinose solution was added and homogenized in an ultrasonic environment for 15 min to allow the diffusion of silver ions to the capping agent. The reaction was started by addition of 6 mL 0.1 mol L^−1^ NaOH solution (at initial pH of the reaction mixture about 9.5). The temperature of 30 ± 2 °C was kept constant in the ultrasound bath and the reaction was completed within 60 min after the first color appearance. A change of the solution color from colorless to pale brown and subsequently to yellow orange was observed indicating silver nanoparticle formation.

Using the synthesis method described above, an aqueous dispersion of raffinose capped silver nanoparticles was obtained with Ag concentration of 1 × 10^−3^ mol L^−1^. This as-synthesized dispersion was denoted as a stock solution of Ag/Raff NPs, which was kept in a dark glass flask at room temperature and used in the experiments for determination of Cr(VI) without any further surface modification. The nanoparticle dispersion was homogenized by ultrasonic bath for 30 min prior to each experiment.

### 3.3. Characterization of Silver Nanoparticles

The optical properties of Ag/Raff NPs in aqueous dispersion were characterized by UV-Vis spectroscopy. The absorbance spectra were recorded by Evolution 300 spectrophotometer (Thermo Fisher Scientific, Waltham, MA, USA) within the 300–900 nm range using a quartz cuvette with a 1-cm optical path. Double distilled water was used as a reference sample for background absorption in all measurements. For the purpose of nanoparticle characterization, it was necessary to dilute the as-prepared samples in order to avoid measurement inaccuracy (UV-Vis spectroscopy) or even to enable measurements of zeta potential. Therefore, a dilution of the nanoparticle dispersion to silver concentration of 10 mg L^−1^ was conducted.

The morphology and particle sizes of silver nanoparticles in as-synthesized aqueous dispersion were examined using a transmission electron microscope (TEM JEOL 2100) operating at accelerating voltage of 200 kV. A volume of 5 µL Ag/Raff NPs dispersion was placed on a carbon-covered copper grid for TEM and then air-dried. The size distribution of silver nanoparticles and average nanoparticle diameter were determined from TEM images counting at least 200 particles using imaging software (ImageJ).

The crystal structure of raffinose capped silver nanoparticles was confirmed by selected area electron diffraction (SAED) pattern obtained from TEM observations and X-ray diffraction (XRD) analysis. The XRD patterns of raffinose powder and layer of Ag/Raff NPs, dried on a glass support, were registered using an X-ray powder diffractometer (Siemens D500) equipped with CuKα radiation (λ = 1.54 Å) in 2*θ* ranging from 5° to 85°.

The hydrodynamic diameter of raffinose capped AgNPs was measured by dynamic light scattering (DLS) using a Particle Size Analyzer LB-550 HORIBA equipped with a 5-mW laser diode (650 nm wavelength) and photo-multiplier tube (Photocell detector). Before measurements, the samples were filtered using Inorganic Membrane Filter Anotop 10 (0.2 μm, Whatman) to remove any interfering dust particles. Zeta (ζ) potential of silver nanoparticles was measured with a Zetasizer Nano ZS (Malvern) instrument. The measurement was replicated three times per sample in order to check the reproducibility of the obtained results.

### 3.4. Colorimetric Detection of Cr(VI)

The colorimetric detection of Cr(VI) ions via raffinose capped silver nanoparticles was conducted as follow: an aliquot of 200 μL Ag/Raff NPs stock solution and 150 μL 5 × 10^−3^ mol L^−1^ ascorbic acid solution were mixed into a small quartz cuvette and homogenized on a Vortex for 30 s; 1650 μL Cr(VI) standard solution with varying concentrations and pH 3.8 (achieved by HCl) were added and re-homogenized on Vortex for another 30 s. After the optimal contact time, the UV-Vis spectrum of the final mixture was recorded in the wavelength range of 300–800 nm. Each set of experiments was carried out in triplicates and average values of the results and standard deviations were calculated.

In order to investigate the sensitivity of the colorimetric assay towards other ions, raffinose capped silver nanoparticles were allowed to interact separately under the same conditions with 50 μmol L^−1^ solutions of alkali (Na^+^, K^+^), alkaline earth (Mg^2+^, Ca^2+^), Pb^2+^ and transition-metal ions (Cu^2+^, Zn^2+^, Cd^2+^, Fe^3+^, Co^2+^, Ni^2+^, Cr^3+^). The resulting mixtures were monitored by UV-Vis absorption spectroscopy.

### 3.5. Analytical Procedure for Cr(VI) Determination in Water

A water sample of 20 mL was filtered through a 0.45 μm filter and acidified with 1 mol L^−1^ HCl until pH 3.8. A sample aliquot of 1650 μL was transferred into quartz cuvette, where a pre-mixed solution of 200 µL stock solution of Ag/Raff NPs and 150 μL ascorbic acid solution (5 × 10^−3^ mol L^−1^) was added and the resulting mixture was stirred on Vortex for 30 s. After the optimal contact time of 5 min, the UV-Vis absorbance was measured at λ_max_ of 411 nm.

## 4. Conclusions

In conclusion, we have established a rapid, easy, and time-efficient sensing strategy for the selective quantification of Cr(VI) using raffinose capped silver nanoparticles as a simple optical LSPR based probe. Careful optimization of sensor response time permits reliable determination of Cr(VI) in the presence of excess concentrations of Cr(III) without any preliminary separation of both species. The interaction of Ag/Raff NPs with in situ reduced Cr(VI) (ascorbic acid as a reducing agent) leads to a color change of Ag/Raff NPs dispersion in the first 5 min of the contact time, which indicates their aggregation with a corresponding decrease of LSPR band intensity and the appearance of a shoulder band at longer wavelengths. To date, no reports have existed for the speciation of chromium using unmodified silver nanoparticles, which allows direct selective determination of Cr(VI).

## Figures and Tables

**Table 1:** Selective determination of Cr(VI) in the presence of Cr(III) in model solutions and water samples (5 parallel determinations).

Sample	Added Cr(III), µmol L^−1^	Added Cr(VI), µmol L^−1^	Found Cr(VI), µmol L^−1^	Recovery, %
Model solution	10	10	9.7 ± 0.2 ^1^	97
Model solution	50	10	9.8 ± 0.2 ^1^	98
Model solution	100	10	9.7 ± 0.2 ^1^	97
River water (Tundja)	50	8	8.2 ± 0.2 ^2^	102
Mineral water (Bankya)	50	8	8.1 ± 0.3 ^2^	101
Mineral water (Gorna Banya)	50	8	7.9 ± 0.2 ^2^	99

^1^ quantification against external calibration curve; ^2^ quantification using standard addition.

**Table 2:** Comparative analysis of ground waters from region of Yambol (5 parallel determinations). In all cases, *t*_calc_ is below *t*_crit_ = 2.73 (95%, 9).

Sample	Total Cr, µg L^−1^ ICP-MS; Mean ± SD	Cr(VI), µg L^−1^ Proposed Method; Mean ± SD	Cr(VI), µg L^−1^ ISO; Mean ± SD	*t*_calc_^1^
Sample 1	346 ± 9	318 ± 16	325 ± 19	0.9
Sample 2	576 ± 11	521 ± 20	532 ± 21	1.2
Sample 3	126 ± 4	67 ± 3	64 ± 2	2.1

^1^ Student’s *t* test.

## Data Availability

Not applicable.

## Sample Availability

Samples of the compound (raffinose capped silver nanoparticles in aqueous dispersions) are available from the authors.
